# Five Years Allograft Survival of Renal Transplants: A Single Center Study

**Published:** 2011-10-01

**Authors:** E Ghanei, M R Razaghi, M Homayouni

**Affiliations:** 1 Department of Kidney Transplantation, Shohadae-Tajrish Hospital, Shahid Beheshti University of Medical Sciences, Tehran, Iran; 2 Department of Internal Medicine, Shohada-e-Tajrish Hospital,Shahid Beheshti University of Medical Sciences, Tehran, Iran

**Keywords:** Graft survival, Patients survival, Kidney transplantation, Iran

Dear Editor,

Renal transplantation is the treatment of choice for vast majority of patients with end-stage renal disease.1 Marked improvements in early graft survival and graft function have resulted in kidney transplantation being a cost-effective alternative to dialysis.[2] Among the most age groups and etiologies of end-stage renal disease, renal transplantation prolongs patient lifespan.[3][4] Accordingly in this study, short and long-term follow ups of patients undergoing kidney transplant were evaluated in a thirteen-year cohort study.

In this cohort study, 306 consecutive patients (age from 11 to 66 years) with end -stage renal disease (ESRD) between 1995 and 2008 attending to our institution were recruited. They were selected in a census manner. Procedure of surgery was usually performed consecutively by the same surgical team. Immunosuppressive regimens were based on a similar protocol. All patients were followed up routinely in our center. The Shahid-Beheshti Ethical Committee approved this study and the study was performed in accordance with the declaration of Helsinki.

The variables included age, sex, race, BMI, social class, alive or dead status of kidney donor, hypertensive status, BUN and creatinine in one month, one year, and five years after kidney transplantation. The exact time of transplantation was considered to be the initial event, and irreversible loss of renal allograft (when the patients needed regular dialysis again) was defined as the end-point event. Data were collected through review of hospital and nephrology clinic records. The organ survival and return to regular dialysis were assessed and determined by nephrologists in follow up records in the clinic.

After testing for normal distribution by Kolmogorov-Smirnov test, continuous data were compared by t test and categorical data were compared by the Chi-Square and Fisher Exact tests. For analyzing survival rate, Kaplan-Meier method was applied .Survival data were analyzed by running SPSS software (Version 16, Chicago, IL, USA). Differences with a p<0.05 in analysis were considered as statistically significant.

The mean age of the patients was 35±24 years (range=11-66 years), 126 subjects (41.18%) were female and 180 patients (58.82%) were male. Two patients were on peritoneal dialysis, 28 cases had no history of dialysis, and the other subjects were on hemodialysis, before the kidney transplantation was performed. All patients were on immuno-suppressors after the transplantation. 290 out of 306 cases of donors were living and 16 out of 306 were cadavers. The mean duration of follow up in this study was 50±30.6 months.

The one-month graft and patients survival rate were 93.5±0.5% and 99.3±0.4% respectively. There were 20 cases of graft loss (6.53%) in the first month that six cases were for accelerated rejection and fourteen were for venoarterial thrombosis. The one-year graft and patients survival rate were 86.89±0.6% and 96.54±0.3% respectively. The five-year graft and patient's survival rate were 81±0.8%and 85±0.5% respectively. The recorded BUN and creatinine levels were shown in Table 1 after one month, one year, and five year follow ups.

Kidney transplantation is the treatment of choice in patients with end-stage renal disease because it results in better survival and quality of life. It has been reported that racial and socioeconomic variations may influence the results of kidney transplantation. Hence performing studies in different populations seems necessary.[5][6] In Vergoulas study, it was seen that the patient survival at 1 year was 93.1%-100%. Graft survival at 1 and 5 years was 100%. We had a relatively similar frequency in our studies.[7]

Totally, according to the obtained results in this study and comparison with previous similar studies, it may be concluded that our patients had a similar prognosis compared with previous reports. It is recommended to perform more studies to obtain more definite results.

**Table 1:** BUN and creatinine levels after 1 month, 1 year, and 5 years.

**Time**	**BUN (mg/dl)**		**Creatinine (mg/dl)**	
	**Range**	**Mean**	**Range**	**Mean**
After 1 month	6-46	28±10.5	0.5-2.6	1.1±0.5
After 1 year	11-64	30±14.3	0.7-2.8	1.3±0.6
After 5 years	15-78	38±26.5	0.8-3.2	1.56±1.8
